# Implementation of an Automated Antibiotic Time-out at a Comprehensive Cancer Center

**DOI:** 10.1093/ofid/ofae235

**Published:** 2024-04-25

**Authors:** Frank P Tverdek, Samuel L Aitken, Victor E Mulanovich, Javier Adachi, Cai Wu, Sherry S Cantu, Patrick M McDaneld, Roy F Chemaly

**Affiliations:** Division of Pharmacy, The University of Texas MD Anderson Cancer Center, Houston, Texas, USA; Division of Pharmacy, The University of Texas MD Anderson Cancer Center, Houston, Texas, USA; Center for Antimicrobial Resistance and Microbial Genomics, UTHealth McGovern Medical School, Houston, Texas, USA; Department of Infectious Diseases, Infection Control, and Employee Health, The University of Texas MD Anderson Cancer Center, Houston, Texas, USA; Department of Infectious Diseases, Infection Control, and Employee Health, The University of Texas MD Anderson Cancer Center, Houston, Texas, USA; Division of Pharmacy, The University of Texas MD Anderson Cancer Center, Houston, Texas, USA; Department of Infectious Diseases, Infection Control, and Employee Health, The University of Texas MD Anderson Cancer Center, Houston, Texas, USA; Division of Pharmacy, The University of Texas MD Anderson Cancer Center, Houston, Texas, USA; Department of Infectious Diseases, Infection Control, and Employee Health, The University of Texas MD Anderson Cancer Center, Houston, Texas, USA

**Keywords:** antimicrobial stewardship, cancer, carbapenem, smart systems, vancomycin

## Abstract

**Background:**

Antimicrobial stewardship programs can optimize antimicrobial use and have been federally mandated in all hospitals. However, best stewardship practices in immunocompromised patients with cancer are not well established.

**Methods:**

An antimicrobial time out, in the form of an email, was sent to physicians caring for hospitalized patients reaching 5 days of therapy for targeted antimicrobials (daptomycin, linezolid, tigecycline, vancomycin, imipenem/cilastatin, meropenem) in a comprehensive cancer center. Physicians were to discontinue the antimicrobial if unnecessary or document a rationale for continuation. This is a quasi-experimental, interrupted time series analysis assessing antimicrobial use during the following times: period 1 (before time-out: January 2007-June 2010) and period 2 (after time-out: July 2010-March/2015). The primary antimicrobial consumption metric was mean duration of therapy. Days of therapy per 1000 patient-days were also assessed.

**Results:**

Implementation of the time-out was associated with a significant decrease in mean duration of therapy for the following antimicrobials; daptomycin: −0.89 days (95% confidence interval [CI], −1.38 to −.41); linezolid: −0.89 days (95% CI, −1.27 to −.52); meropenem: −0.97 days (95% CI, −1.39 to −.56); tigecycline: −1.41 days (95% CI, −2.19 to −.63); *P* < .001 for each comparison. Days of therapy/1000 patient-days decreased significantly for meropenem (−43.49; 95% CI, −58.61 to −28.37; *P* < .001), tigecycline (−35.47; 95% CI, −44.94 to −26.00; *P* < .001), and daptomycin (−9.47; 95% CI, −15.25 to −3.68; *P* = .002).

**Discussion:**

A passive day 5 time-out was associated with reduction in targeted antibiotic use in a cancer center and could potentially be successfully adopted to several settings and electronic health records.

## Teaser

Implementation of a fully automated antimicrobial time-out at day 5 from starting antibiotics at a comprehensive cancer center was associated with decreased utilization of targeted broad-spectrum antimicrobial agents. Such a system is sustainable, low maintenance, and appears to be of high yield.

## INTRODUCTION

Antimicrobial stewardship programs (ASPs) can optimize antimicrobial use, curtail resistance rates, and reduce health care costs [1–4]. Hospital accreditation entities, including The Joint Commission, have promulgated standards related to antimicrobial stewardship in line with proposed mandatory Centers for Medicare and Medicaid Services conditions of participation [5]. These standards and U.S. Centers for Disease Control and Prevention (CDC) antimicrobial stewardship toolkit propose an antimicrobial time-out as a key element of any ASP [5, 6]. However, the literature on antibiotic time-outs is limited and somewhat mixed [7–9]. There is also a dearth of antimicrobial stewardship literature in immunocompromised patients [10]. An antibiotic time-out has been a major component of our ASP at our institution since 2010, predating the Joint Commission/Centers for Medicare and Medicaid Services suggestions. Here, we provide a historical perspective on the growth of antimicrobial stewardship at our institution and report our experience with the use of an antibiotic time-out (ABX).

### Historical Perspective on Antimicrobial Stewardship at the University of Texas MD Anderson Cancer Center

The first attempt at antimicrobial stewardship at our institution, in the late 1990s, employed vancomycin restriction via order form along with aggressive infection control measures to reduce vancomycin-resistant enterococci (VRE) infections [11]. However, because of conversion from written to electronic medication order entry, the use of the order form was discontinued. In 2005, we identified carbapenem use exceeding 6 days as a risk factor for multidrug resistant (MDR) *Pseudomonas aeruginosa* [12]. Subsequently, an educational campaign focusing on judicious use of carbapenems was associated with reductions in use of carbapenems and decreased rates of nosocomial MDR *P aeruginosa* infections [13]. Over time, the associated benefits diminished (unpublished internal data). Therefore, in 2007 a quality improvement project targeted unnecessary carbapenem and vancomycin use in our intensive care units (ICUs). A stewardship team comprising an infectious diseases (ID) physician and a physician assistant provided direct feedback at day 7 to the primary team physician regarding carbapenem and vancomycin therapy. The project demonstrated a decreased overall use of vancomycin and carbapenems as well as an associated decrease in nosocomial VRE and MDR *P aeruginosa* infections (unpublished internal data). However, on completion of the pilot study, the intervention was phased out because of its resource-heavy nature. Therefore, a less labor-intensive modality was sought. To that end, a new strategy was designed to implement an alerting system to prompt an antimicrobial time-out.

## METHODS

### Hospital Description

Our institution, a National Cancer Institute-designated comprehensive cancer center, had 650 beds at the time of the intervention. Roughly half of the admitted patients at any given time have underlying hematologic malignancy or are undergoing hematopoietic cell transplantation, with the remainder comprising various medical and surgical oncology patients with solid tumors. There are approximately 50 ICU beds in service. The electronic health record was custom built and allowed for electronic medical charting as well as electronic order prescribing.

Hospital services are organized by type of underlying malignancy and consist of an oncologist as the attending physician supported by advanced practice providers (physician assistants and nurse practitioners) and occasionally medical fellows. Most medical oncology services include a clinical pharmacist with ability to prescribe noncontrolled substance medications in the inpatient setting under collaborative practice agreements. There were 4 dedicated inpatient ID consultation services with a total daily census between 70 and 110 patients. Two ID clinical pharmacists were members of the ID consult services.

### Stewardship Intervention

Starting in November 2010, an email alert was sent by an administrative assistant in the hospital quality department to all attending physicians for hospitalized patients reaching 5 days of continuous therapy of targeted antimicrobials (daptomycin, linezolid, tigecycline, vancomycin, imipenem/cilastatin, meropenem). Day 5 was calculated through a daily query of the pharmacy database of all inpatient active antimicrobials. An algorithm defined a drug reaching day 5 as receiving at least 2 dose of the targeted drug on each day for 4 consecutive days with no more than 48 hours between doses and with an active order present on the fifth day. The email was meant to be informative, and no specific response outside of modification of antibiotic duration, as deemed appropriate by the treating physician, was expected. No antimicrobials were restricted by other means at the time this intervention was implemented. Services targeted by this intervention were the leukemia, hematopoietic cell transplantation, and lymphoma/myeloma teams (including all patients in the ICU) because they accounted for >80% of targeted antimicrobial use at the time. This program was preceded by an educational campaign, delivered to targeted departments’ monthly meetings and consisting of short lectures (∼15 minutes) on the logistics of the program and importance of judicious use of antimicrobials These began in July 2010 to generate awareness of the initiative. This modality of passive time-out on day 5 was consistent throughout the intervention period. However, because it was an iterative quality improvement process, there were multiple changes made in the manner the alerts manifested. Feedback from recipients of the alerts, as well as periodic compliance assessments, drove the changes. There were no other major antimicrobial stewardship initiatives during this period or relevant drug shortages.

**Figure 1.**
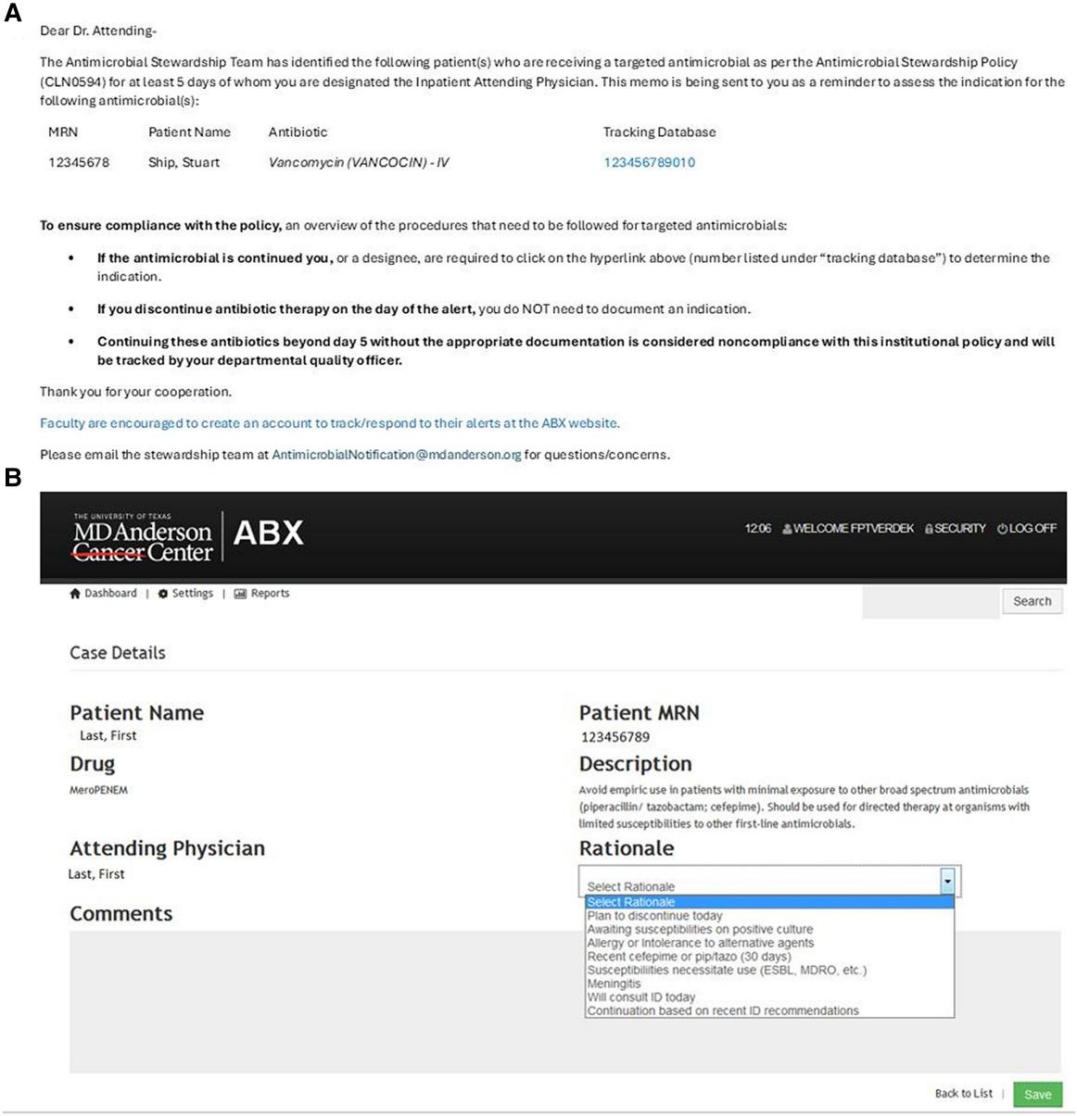
Current ABX user interface. Generic email template sent to the attending physician (*A*); web user interface after following hyperlink (*B*).

In March 2012, a duplicate of the time-out email was sent to the infectious diseases consult physician for any patient with an infectious diseases consult. Throughout the intervention, physicians receiving the alerts were instructed to discontinue the antimicrobial if unnecessary or to document the reason(s) for continuation, using a predefined set of rationales in the electronic medical record, with an allowance for free-text responses if needed. Discontinuation and rationales were periodically monitored by the ASP physician champion and the ID clinical pharmacists. The alert itself acted as a reminder and was not coupled with a “hard stop” of these targeted antibiotics. In June 2014, a custom-built secure web interface (ABX) fully automated this process. The ABX system linked the pharmacy database and the remainder of our electronic medical record to identify duration of therapy, the attending physician, and ID consultation. Personalized emails were sent automatically at 6:30 Am each day and directed the primary inpatient physician on record to a user-friendly web interface (Figure 1) to document the rationale for use in the online database. ID-consulted patient alerts were only sent to ID physicians who were required to respond in the same manner as primary team physicians.

### Antimicrobial Use

This is a quasi-experimental, interrupted time series analysis assessing antimicrobial use during the following time periods: period 1 (before time-out: January 2007-June 2010 inclusive) and period 2 (after time-out: July 2010-March 2015). The starting point for period 2 was chosen based on the start of the educational campaign as described previously, whereas the end of period 2 was chosen based on further major program changes. As the intervention targeted excess duration of therapy, the primary antimicrobial consumption metric was mean duration of therapy, defined as the number of antibiotic days divided by the total number of antibiotic courses initiated. Mean, rather than median, was chosen specifically as a metric sensitive to outliers. In addition, days of therapy per 1000 patient days (DOT/1000 PD) were also assessed. Each metric was assessed on a monthly basis throughout both periods and were routinely reported to the ASP team and hospital administration. The targeted antimicrobials serve as the intervention group and nontargeted antimicrobials (ceftazidime, cefepime, ertapenem, and piperacillin-tazobactam) served as a contemporaneous control group.

Segmented ordinary least squares regression analysis was performed for each drug/consumption metric assessed as has previously been described [14, 15]. For each combination, a single ordinary least squares regression model incorporating slope parameters for consumption before and after July 2010 as well as an intercept for July 2010 (ie, the predicted value in July 2010 without intervention) was created. The reported differences in consumption correspond to a linear combination of the parameter estimates as predicted for July 2010. Thus, each comparison allows for an assessment of immediate change in consumption (intercept) and long-term change in consumption (slope).

### Healthcare-Associated Infections

Healthcare-associated infections (HAI; MDR *P aeruginosa*, VRE, extended-spectrum β-lactamase [ESBL] producers, carbapenem-resistant Enterobacterales [CRE], and *Clostridium difficile* infection [CDI]) were identified in the infection control database as per CDC/National Healthcare Safety Network surveillance definitions and guidelines [16–18]. Standard incidence rates were calculated and assessed annually for each organism. MDR *P aeruginosa* was defined as *P aeruginosa* resistant to either meropenem or imipenem or at least 3 of the following groups: ceftazidime/cefepime or piperacillin/tazobactam or ciprofloxacin/levofloxacin, according to definitions in use during this time by the infection control program. CDI was defined as diarrhea and a positive CDI stool assay (enzyme-linked immunosorbent assay for toxin B until 2015, nucleic acid amplification testing for the toxin B thereafter). Poisson regression, adjusting for the number of patient-days in a calendar year, was used to describe secular trends in HAI rates and reported as the incidence rate ratio (IRR).

All analyses were conducted using Stata v14.1 (StataCorp LP, College Station, TX).

## RESULTS

### Antimicrobial Use

**Figure 2.**
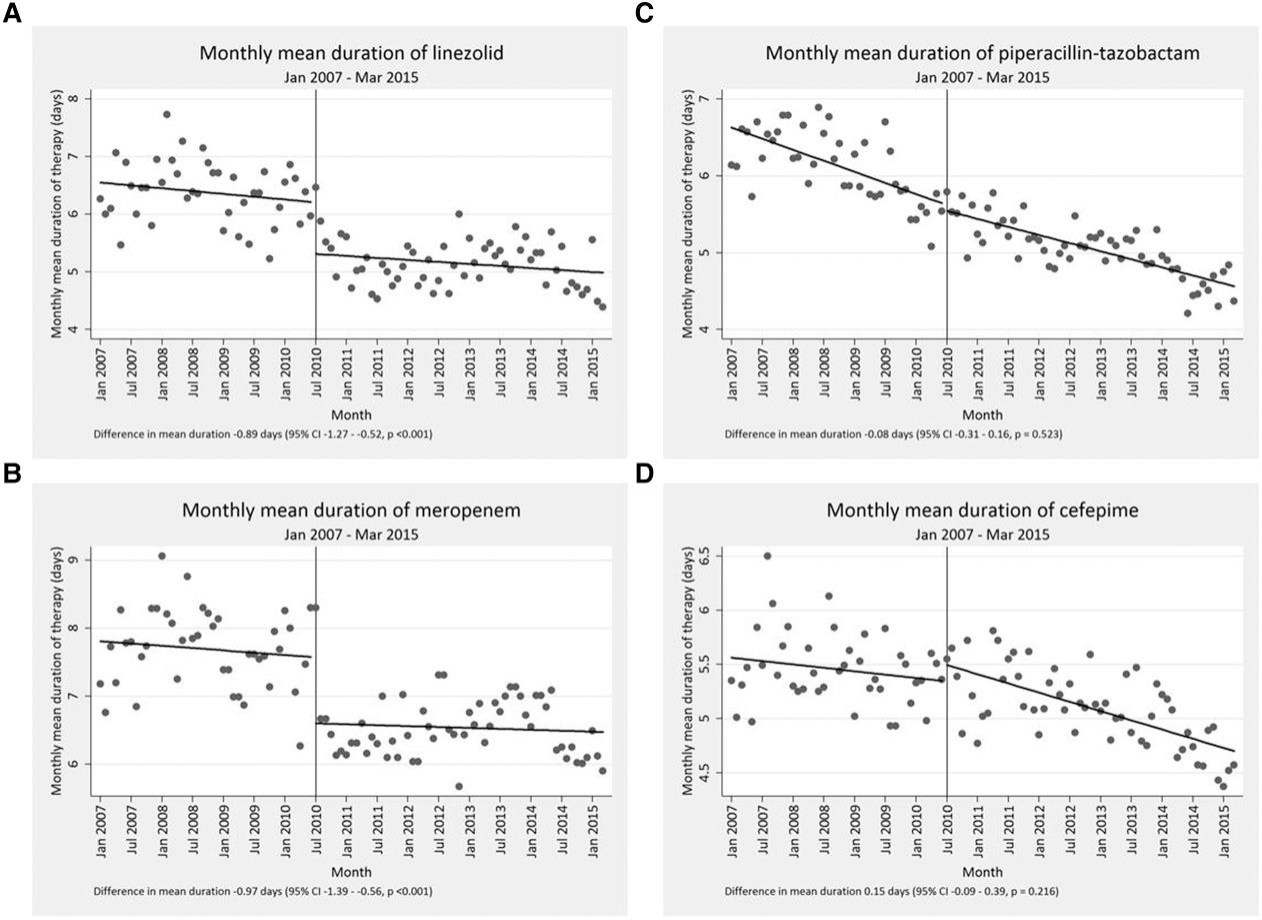
Representative examples of segmented regression analysis for 2 targeted (*A* and *B*) and 2 nontargeted antibiotics (*C* and *D*).

**Figure 3.**
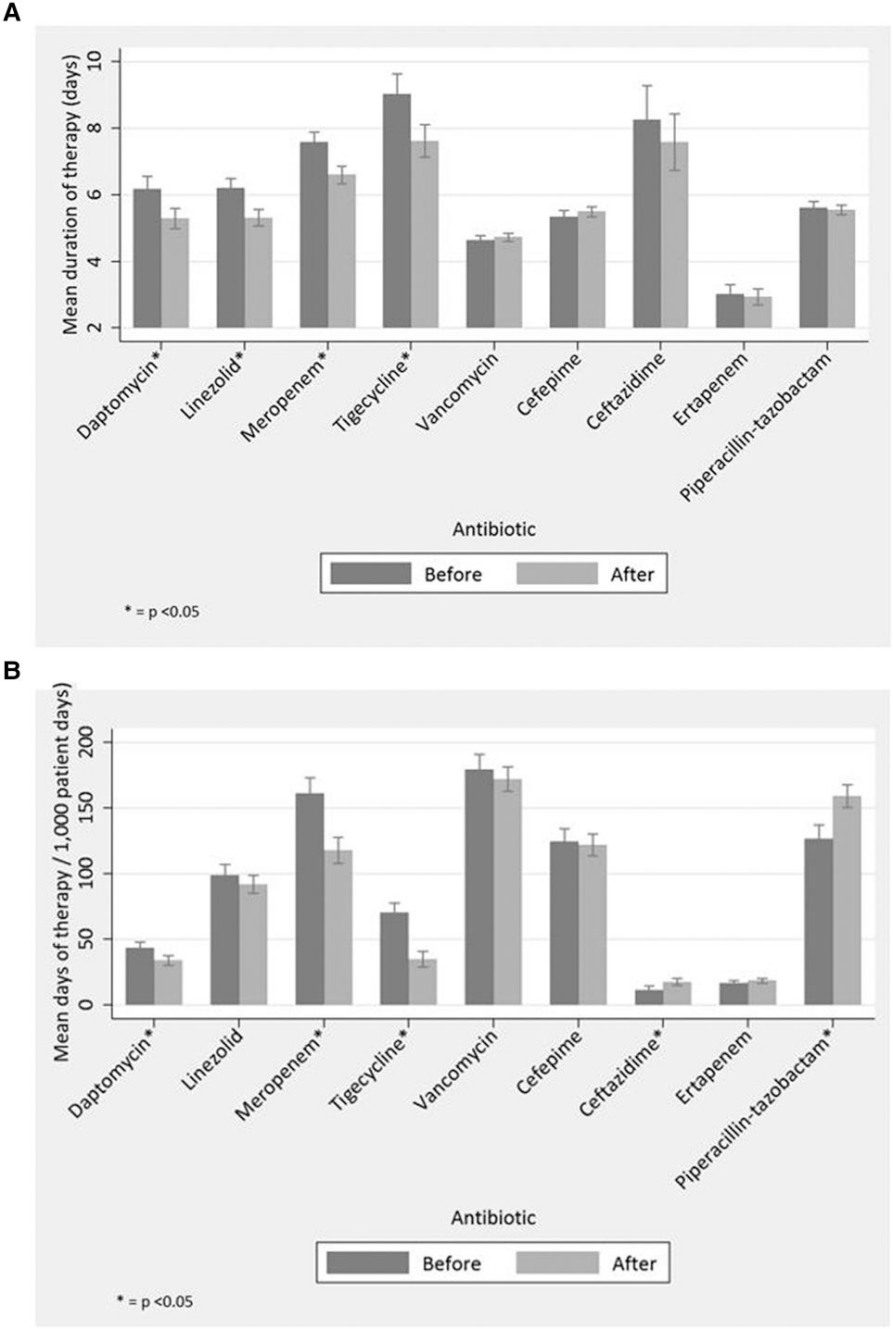
(*A*) Mean duration of therapy before and after start of time-out for targeted (daptomycin, linezolid, meropenem, tigecycline, vancomycin) and nontargeted (cefepime, ceftazidime, ertapenem, piperacillin-tazobactam) antibiotics. Bar heights and corresponding 95% confidence intervals correspond to the July 2010 intercept for the before and after components of the model. (*B*) Days of therapy per 1000 patient days (DOT/1000 PD) before and after start of time-out for targeted (daptomycin, linezolid, meropenem, tigecycline, vancomycin) and nontargeted (cefepime, ceftazidime, ertapenem, piperacillin-tazobactam) antibiotics. Bar heights and corresponding 95% confidence intervals correspond to the July 2010 intercept for the before and after components of the model.

**Figure 4.**
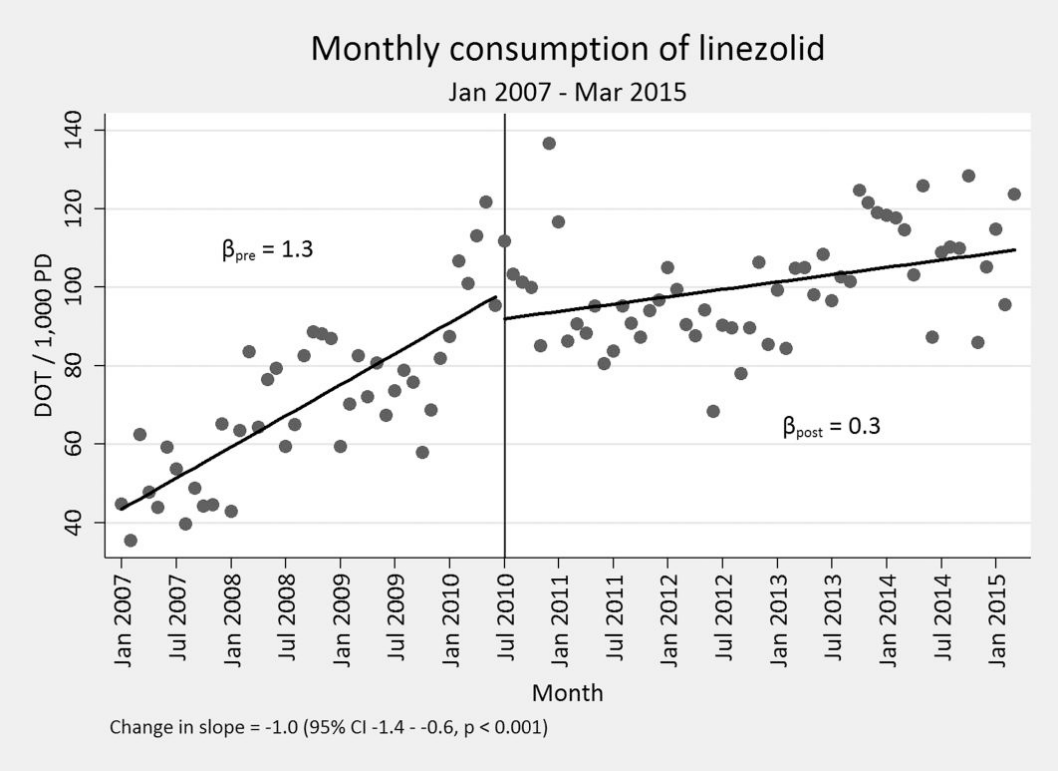
Days of therapy per 1000 patient days (DOT/1000 PD) before and after start of time-out for linezolid.

Overall, the mean duration of therapy for each drug assessed in the study was slowly declining before the start of the intervention. Beyond this general trend, the mean duration of therapy significantly decreased for each of the targeted drugs, with the exception of vancomycin, following implementation of the time-out. This decrease was almost 1 day of mean duration of therapy for daptomycin, linezolid, and meropenem (−0.89; 95% confidence interval [CI], −1.38 to −.41), −0.89 (95% CI, −1.27 to −.52), and −0.97 (95% CI, −1.39 to −.56 respectively). Tigecycline notably had a decrease in excess of 1 day (−1.41 [95% CI, −2.19 to −.63]). Full assessments are presented in Table 1, Figure 2, and Figure 3. Among targeted drugs, vancomycin did not have a significant change (0.09 [95% CI, −.1 to −.29]). These significant reductions in duration of therapy additionally corresponded with both short- and long-term reduction in DOT/1000 PD, with the exception of vancomycin and linezolid (Table 2, Figure 3). Linezolid DOT/1000 PD was dramatically increasing before implementation of the time-out. After intervention, it continued to increase, albeit at a significantly slower rate (Figure 4). There were no significant changes in the mean length of hospital stay in the before and after intervention periods (data not shown).

**Table 1.** Change in Mean Duration of Therapy Following Implementation of Antibiotic Time-out

	Intercept Parameters (Indicative of Immediate Change)				Slope Parameters (Indicative of Long-Term Change)			
Antibiotic	Baseline Value	Calculated Change	95% CI	*P V*alue	Baseline Value	Calculated Change	95% CI	*P V*alue
Targeted antibiotics								
Daptomycin	6.18	−0.89	−1.38 to −.41	<.001	−0.01	0.01	−.01 to .03	.291
Linezolid	6.21	−0.89	−1.27 to −.52	<.001	−0.01	0.00	−.01 to .02	.741
Meropenem	7.58	−0.97	−1.39 to −.56	<.001	−0.01	0.00	−.01 to .02	.669
Tigecycline	9.03	−1.41	−2.19 to −0.63	.001	0.00	−0.01	−.03 to .02	.724
Vancomycin	4.63	0.09	−.10 to .29	.342	−0.01	−0.04	−.01 to .00	.313
Nontargeted antibiotics								
Cefepime	5.34	0.15	−.09 to .39	.216	−0.01	−0.01	−.02 to −.00	.047
Ceftazidime	8.26	−0.67	−2.00 to .66	.322	0.04	−0.03	−.08 to .15	.177
Piperacillin-tazobactam	5.62	−0.08	−.31 to .16	.523	−0.02	0.01	.00 to .02	.139
Ertapenem	3.02	−0.09	−.47 to .29	.656	−0.02	0.03	.02 to .05	<.001

All values correspond to a linear combination of parameter estimates for the before and after values of the segmented regression analysis.

Abbreviation: CI, confidence interval.

**Table 2.** Change in Days of Therapy per 1000 Patient Days (DOT/1000 PD) Following Implementation of Antibiotic Time-out

	Intercept Parameters (Indicative of Immediate Change)				Slope Parameters (Indicative of Long-Term Change)			
Antibiotic	Baseline Value	Calculated Change	95% CI	*P* Value	Baseline Value	Calculated Change	95% CI	*P* Value
Targeted antibiotics								
Daptomycin	43.43	−9.47	−15.25 to −3.68	.002	0.43	−0.23	−.45 to −.02	.032
Linezolid	98.90	−6.94	−17.54 to 3.66	.197	1.32	−1.01	−1.40 to −.62	<.001
Meropenem	161.50	−43.49	−58.61 to −28.37	<.001	1.09	−1.39	−1.95 to −.83	<.001
Tigecycline	70.42	−35.47	−44.94 to −26.00	<.001	0.81	−0.67	−1.02 to −.33	<.001
Vancomycin	179.57	−7.38	−22.12 to 7.36	.323	−0.36	−0.18	−.72 to .36	.516
Nontargeted antibiotics								
Cefepime	124.44	−2.32	−15.18 to 10.54	.721	−0.15	0.60	.13-1.08	.013
Ceftazidime	11.11	6.48	2.05 to 10.90	.005	0.12	0.01	−.15 to .17	.897
Piperacillin-tazobactam	126.66	32.51	18.74 to 46.27	<.001	−0.61	0.18	−.32 to .69	.477
Ertapenem	16.61	1.97	−.79 to 4.73	.160	0.27	−0.14	−.24 to −.04	.008

All values correspond to a linear combination of parameter estimates for the pre- and post- values of the segmented regression analysis.

Abbreviations: CI, confidence interval; DOT/1000 PD, days of therapy per 1000 patient-days.

In contrast to the targeted antimicrobials, the mean duration of therapy did not significantly change, short-term, for the nontargeted antibiotics. However, a long-term decrease was seen for cefepime and long-term increase was seen for ertapenem (Table 2, Figure 3). The DOT/1000 PD rose significantly for ceftazidime and piperacillin-tazobactam, whereas long term-increases and reduction were observed for cefepime and ertapenem, respectively.

### Healthcare-Associated Infections

**Figure 5.**
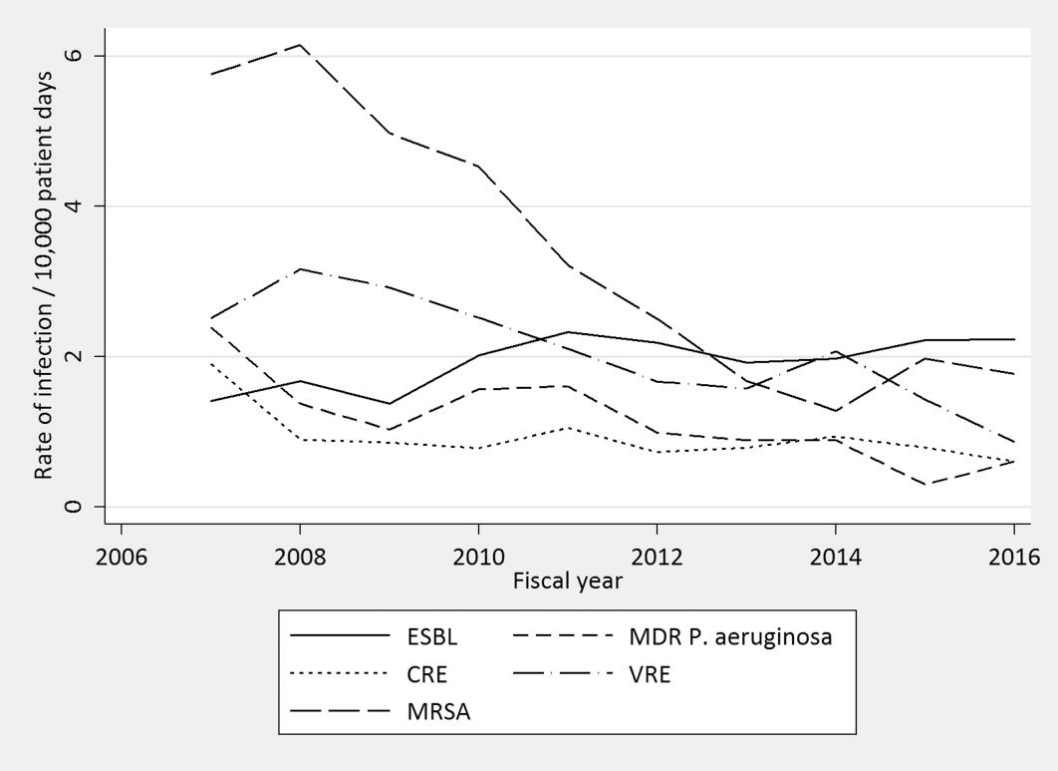
Rates of health care-associated infections from fiscal year 2007 to fiscal year 2016. CRE, carbapenem-resistant Enterobacterales; ESBL, extended spectrum β-lactamase; MDR, multidrug-resistant; MRSA, methicillin-resistant *Staphylococcus aureus*; VRE, vancomycin-resistant Enterococcus.

After time-out implementation, significant reduction in HAI rates were observed for methicillin-resistant *Staphylococcus aureus* (IRR, 0.84; 95% CI, .81–.86; *P* < .001), VRE (IRR, 0.90; 95% CI, .87–.93; *P* < .001), MDR *P aeruginosa* (IRR, 0.86; 95% CI, .82–.91; *P* < .001), and CRE (IRR, 0.93; 95% CI, .88–.98; *P* = .004). A significant increase in ESBL-producing Enterobacterales infections was observed during the same period (IRR, 1.04; 95% CI, 1.01–1.08; *P* = .024). Because of changes in testing methods for CDI during this time period, formal statistical testing was not performed for this HAI. However, rates initially declined and then sharply increased following introduction of nucleic acid amplification testing. HAI rates are presented in Figure 5.

## DISCUSSION

A passive email-based antibiotic time-out on day 5 of therapy intervention was associated with a decrease in mean duration of therapy for a number of targeted broad-spectrum antimicrobials in hospitalized patients with underlying hematologic malignancies. This decrease in mean duration of therapy coincided with reduction in overall use of antimicrobials as evidenced by a decrease in DOT/1000 PD. Consistent with this change being caused by the intervention, no changes in the mean duration of therapy for nontargeted antibiotics was seen; however, it is worth noting that overall use of piperacillin-tazobactam and ceftazidime significantly increased following the intervention.

The desired effect of reduction in mean duration of therapy for targeted antibiotics was observed following implementation of the time-out. Because of the nonrandomized nature of the study, a causal relationship could not be ascertained. However, the consistency of the findings among targeted antibiotics and lack of observed positive effect in nontargeted antibiotics favor the potential contribution of the intervention. The lack of decrease in the mean duration of therapy for vancomycin paradoxically supports the effect of the intervention because the mean duration of therapy was already less than 5 days before the time-out was implemented and a further decrease in duration was unlikely.

Although our goal of reducing the mean duration of therapy for targeted agents was realized, this coincided with significant increase in the use of piperacillin-tazobactam and ceftazidime. This “squeezing the balloon” phenomenon has previously been described in response to a hospital-wide restriction of cephalosporins [19]. This phenomenon at our institution was likely a drive away from meropenem to piperacillin-tazobactam and ceftazidime. This effect was felt to be a favorable, if not desired, outcome. Because piperacillin-tazobactam and ceftazidime are the most reliably active anti-pseudomonal β-lactams at our institution, this shift correlates with the desired spectrum of activity in patients with cancer. Cefepime and ertapenem use remained roughly similar immediately following implementation of the intervention, but long-term changes were observed, correlating with therapy shift away from meropenem. It is important to note that inpatient length of stay remained largely consistent throughout the study period.

We demonstrated that an antibiotic time-out can be implemented in a large institution as a passive intervention that requires minimal daily time commitment from the ASP team. As antimicrobial time-outs are being considered, we provide data on the feasibility and sustainability of this intervention [1]. In 1 study, the use of a twice-weekly antibiotic checklist by medical residents was associated with a significant decrease in moxifloxacin use and decreased antibiotic costs; however, the process was time consuming and would likely be not sustainable [14]. A more recent study targeting carbapenems on day 3 of therapy and with potential of ID intervention, demonstrated a reduction in carbapenem DOT [19]. Of note, this process required ASP review of patients and ID consult in the event the drug went beyond day 3. In contrast, our intervention was largely passive and conducive to limited ASP resources. This is relevant for institutions with limited ASP personnel and resources or those with competing demands for the ASP's time.

A unique distinction of our intervention was the involvement of the ID physicians in addition to the general practitioners because most often ASPs are an extension of the ID department's responsibilities. Early in our process, we found that up to 70% of patients with prolonged antimicrobial therapy had an active ID consultation. Thus, the decision to expand the time-out solely to ID physicians following these patients was deemed essential to affect antimicrobial use and to lead by example. We observed improved engagement by the primary team physicians when ID physicians were held to the same level of scrutiny. This highlights the importance of understanding the institutional context and assessing the relative contributions of all clinicians to unnecessary antimicrobial duration.

Because of the multiple contributing factors associated with antimicrobial resistance and HAI, the role of antimicrobial stewardship in limiting antimicrobial resistance has been difficult to prove. As carbapenem use highly correlates with carbapenem resistant *P aeruginosa*, the temporal relationship of reduction in the rates of MDR *P aeruginosa* and consumption of meropenem over time suggest a strong association [20, 21]. Furthermore, another study showed a significant decline in carbapenem-resistant *P aeruginosa* following implementation of a β-lactam restriction program [22]. The relationship of our intervention to other HAIs is more difficult to assess. For example, the rates of methicillin-resistant *S aureus* have declined nationwide because of a variety of factors [23] and the reported CRE rates in our institution were already low and declined slightly over the period [24]. Interestingly, the rates of ESBL-producing Enterobacterales infections slightly increased over this time period, in line with the increased prevalence across the United States [25–27].

There are limitations to our analysis. We were unable to control for confounding factors during the intervention period including possible changes in staff, escalation of infection control measures and other unknown factors that may have drove the associations seen in antimicrobial use or resistance and the intervention. During the time of intervention, the implementation of ultraviolet light disinfection was implemented in our hospital as well as incremental improvements in hand hygiene initiatives. Second, we did not address the safety of discontinuation of antimicrobials in this immunocompromised patient population. However, there was no penalty for continuation of the targeted antimicrobials for appropriate indications.

In summary, we demonstrated reduction in targeted antibiotic use in a cancer center through the implementation of passive email-based antimicrobial time-out. This time-out is a fully automated process, freeing up the antimicrobial stewardship team to engage in other activities. Such a program could be successfully adopted to several settings and electronic health records.

## Notes

***Acknowledgments.*** Thank you to Bacardi Bryant, Kevin Brammer, and Stephen Lim for creating the ABX alerting system.

***Patient Consent.*** This project was approved by the MD Anderson institutional quality improvement advisory board and all metrics assessed in this report were part of routine business operations, therefore, the need for institutional review board approval and patient consent was waived.

***Financial support*.** None.
